# Acoustic trapping of microbubbles in complex environments and controlled payload release

**DOI:** 10.1073/pnas.2003569117

**Published:** 2020-06-22

**Authors:** Diego Baresch, Valeria Garbin

**Affiliations:** ^a^Department of Chemical Engineering, Imperial College London, London SW7 2AZ, United Kingdom;; ^b^University of Bordeaux, CNRS, Institut de Mécanique et d’Ingénierie (I2M), UMR 5295, F-33405 Talence, France;; ^c^Department of Chemical Engineering, Delft University of Technology, 2629 HZ Delft, The Netherlands

**Keywords:** acoustical tweezers, acoustic force, microbubbles, micromanipulation

## Abstract

Contactless manipulation of microparticles using acoustic waves holds promise for applications ranging from cell sorting to three-dimensional (3D) printing and tissue engineering. However, the unique potential of acoustic trapping to be applied in biomedical settings remains largely untapped. In particular, the main advantage of acoustic trapping over optical trapping, namely the ability of sound to propagate through thick and opaque media, has not yet been exploited in full. Here we demonstrate experimentally the use of the recently developed technique of single-beam acoustical tweezers to trap microbubbles, an important class of biomedically relevant microparticles. We show that the region of vanishing pressure of a propagating vortex beam can confine a microbubble by forcing low-amplitude, nonspherical, shape oscillations, enabling its full 3D positioning. Our interpretation is validated by the absolute calibration of the acoustic trapping force and the direct spatial mapping of isolated bubble echos, for which both find excellent agreement with our theoretical model. Furthermore, we prove the stability of the trap through centimeter-thick layers of bio-mimicking, elastic materials. Finally, we demonstrate the simultaneous trapping of nanoparticle-loaded microbubbles and activation with an independent acoustic field to trigger the release of the nanoparticles. Overall, using exclusively acoustic powering to position and actuate microbubbles paves the way toward controlled delivery of drug payloads in confined, hard-to-reach locations, with potential in vivo applications.

## Significance

Remote positioning and activation of drug-loaded microparticles in vivo are a central pursuit of nanomedicine. Optical, magnetic, and acoustic fields have been used to position and sort microparticles in vitro, but the translation in vivo remains challenging. Because sound can be transmitted centimeters deep into opaque media, acoustical tweezers are considered an ideal candidate for in vivo contactless manipulation. Here we use a single-beam acoustical trap to manipulate microbubbles in three dimensions through materials that mimic biological tissues. We establish the trapping mechanism, which is strikingly different from conventional acoustic trapping of bubbles. We also demonstrate controlled release from nanoparticle-loaded microbubbles with an independent acoustic trigger. We thus lay the foundations for biomedical applications of single-beam acoustical tweezers.

Contactless manipulation techniques are important tools in biophysics and the life sciences, making it possible to remotely position and control biomolecules, viruses, and cells in vitro, using for instance optical, electric, or magnetic fields (1–5). Optical tweezers offer the highest degree of dexterity and spatial resolution but the high intensity they require is not suited for their use in a biomedical context that typically involves bulky, opaque, and fragile environments. Acoustic-based techniques have emerged as powerful tools covering several length scales for applications ranging from cell sorting (6) and biomolecular force spectroscopy (7) to cell patterning and tissue engineering (8, 9), with appealing characteristics of ultrasound such as abundant forces at low incident powers and deep penetration into complex and absorbing media like tissue (10, 11).

Single-beam acoustical tweezers (12) have overcome the limitations of previous acoustic manipulation schemes, which were hindered by the low spatial resolution obtained with pressure nodes and antinodes in highly controlled geometries and their lack of selectivity at the single-particle level (6, 13–15). Single-beam acoustical tweezers have been shown to combine fully three-dimensional (3D), pinpoint manipulation with abundant forces for individual solid elastic particles. Both features are necessary for their potential use in biomedical settings that usually combine complex geometries and flow conditions. Forces reported are in the micronewton range for particles hundreds of micrometers in size (12). Most recently, studies have underscored the potential of acoustical tweezers to be miniaturized in liquids (16) and dynamically controlled in space and time using acoustic holography in midair (17). However, they have only been shown to trap solid particles in air or water, with a finely controlled propagation path in between the trapping beam and the target object. To date, the trapping of most objects of interest in biomedicine, chemistry, and engineering such as cells, microbubbles, or droplets has not been investigated with single-beam acoustical tweezers.

The ability to individually manipulate a large range of object materials with the precision and selectivity of single-beam acoustical traps would enable designing complex procedures; each particle used as an elementary building block for localized chemistry, drug delivery, or assembly in tissue or organoid engineering (9, 18) and the large penetration of ultrasound in tissue make these procedures viable perhaps even in vivo.

In this paper we explore the capabilities of a single-beam acoustical trap to manipulate individual microbubbles, which are an important class of biomedically relevant microparticles (19–23), in crowded and complex environments including through biological tissue phantoms. Bubble trapping at the nodes or antinodes of a standing wave is usually based on their strong periodic change in volume due to external pressure oscillations (14, 24). In contrast, the phase singularity of vortex beams creates a region of vanishing pressure where volume oscillations cannot be forced. To identify the mechanism responsible for trapping of bubbles by single-beam acoustical traps, we used a theoretical model to compute the acoustic radiation force in 3D and compared the predictions with a direct optical calibration of the trapping force and acoustical measurements of isolated bubble echos, providing a direct link to their dynamics. We identify that low-amplitude (linear), nonspherical bubble oscillations (without volume change) enable their full 3D trapping by a strong lateral attractive force and a net axial pushing force, typically several times larger than their buoyancy at moderate acoustic power. We also show that trapped bubbles can be precisely maneuvered through centimeter-thick, tissue-mimicking, opaque-to-light elastic layers immersed in liquids and use this further to assess the influence of the bubble habitat on its dynamics. Finally, we trap nanoparticle-loaded microbubble cargos and observe the directed payload release toward target walls, activated by an independent acoustic trigger driving violent (nonlinear) bubble oscillations. Our fundamental study therefore demonstrates the potential for a wide use of acoustic manipulation for local and noninvasive operations in complex environments and biomedical settings.

## Results

### Trapping Mechanism of a Microbubble in an Acoustic Vortex Beam.

**Fig. 1.**
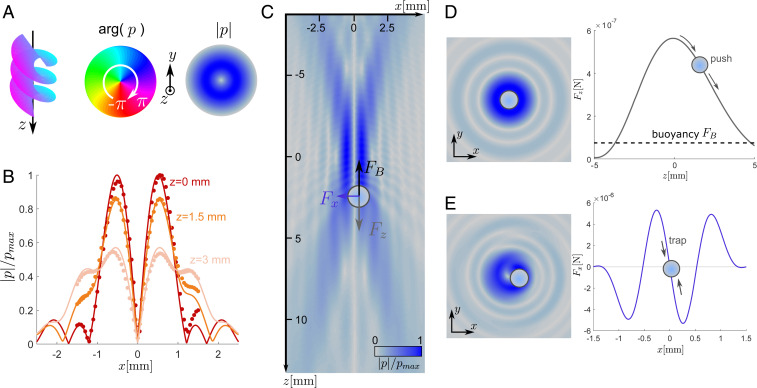
Vortex beam trap for microbubbles. (*A*) The simulated pressure field, |p|, and phase, arg(p), of the trapping vortex beam are shown in the transverse plane (x,y). The phase variation results in a helicoidal wavefront in propagation direction, z, where the pressure must vanish. This is confirmed in *B*, where experimental scans of the pressure field along x for three different axial positions relative to the focal plane z=0, 1.5, and 3  mm are shown in the absence of the bubble. (*C*) The total pressure field surrounding the bubble scattering the beam is simulated in the propagation plane (x,z). (*D*) The same total field seen in the transverse plane (x,y) . It gives rise to the net pushing force $F_{z}$ for a bubble centered on the vortex core (x=y=0). (*E*) Same as *D* for a bubble shifted by a distance x=0.5λ. The total field illustrates how the bubble oscillations distort the incident beam and give rise to a strong lateral trapping force attracting the bubble back toward the vortex core.

Acoustic fields with moderate pressure amplitude drive linear oscillations of the bubble volume, V(t), which can be predicted from a (one-dimensional) model for the bubble radius change (25). In this regime, a bubble in a standing acoustic field, characterized by a pressure gradient $\vec{\nabla }p$, migrates to pressure nodes or antinodes under the action of the primary Bjerknes force (14, 24)

$$\vec{F}=-\left\langle V\left(t\right)\vec{\nabla }p\right\rangle ,$$ [1]

where ⟨⋅⟩ stands for the temporal average over the acoustic period, T=1/f. To establish Eq. **1**, it is also considered that the bubble radius at rest, a, is much smaller than the acoustic wavelength, λ. In the present study, such simplifications do not hold and a general 3D model was used to predict the full dynamic behavior of a bubble in an acoustic vortex beam (26). The phase singularity of the beam, which in our setup varies from −π to π in the transverse plane (x,y), is shown in Fig. 1*A*. It extends all along the propagation axis, z, where a region of vanishing pressure is produced by destructive interference of an incoming helicoidal wavefront. It can thus be expected that volume oscillations vanish for a bubble located precisely at the vortex core. We hypothesized that center-of-mass translations (dipolar) and higher-order nonspherical oscillations (multipolar)—for bubbles with radius a∼λ—would play an important role in the trapping mechanism. A detailed analysis of the dipolar origin of the trapping force for small bubbles is presented in *SI Appendix*.

To test this hypothesis, we first calculated the total acoustic field surrounding a bubble that scatters the incident vortex beam (Fig. 1 *C*–*E*). The acoustic radiation force can subsequently be calculated from the total field, assuming a linear scattering process and bubble dynamics (see *SI Appendix* for details on the calculation). The model predicts that a bubble will be forced by a lateral force, $F_{x}$, toward the propagation axis where it will remain trapped (Fig. 1*E*). Once constrained to the vortex core, a pushing force, $F_{z}$, will operate against the bubble buoyancy, $F_{B}$ (Fig. 1*D*). The situation differs from the behavior of solid particles that are attracted toward the beam focus in all directions (12). In the present case, the combination of the lateral trapping force, the axial pushing force, and the upward buoyancy will allow us to manipulate a microbubble in 3D.

Our experiments confirmed this prediction. The acoustical vortex beam was generated by eight independent transducers driven with the necessary time delays to construct a helicoidal wavefront (see *Methods* and *SI Appendix*, Fig. S2 for further details on the setup and measurements). Fig. 1*B* shows three lateral scans of the acoustic pressure field in the absence of the bubble |p|, at the focal plane (z=0), and downstream (z=1.5 and 3 mm). Comparison with the calculated pressure field indicated that the emission setup used in this study provided a precise spatial control over the phase of the acoustic beam. Movies S1 and S2 show the successful operation of the trap on a bubble with radius a∼200 μm as the trapping beam was displaced. Note that the lateral trapping force shown in Fig. 1*E* is one order of magnitude stronger than its axial counterpart (Fig. 1*D*). Hence, when the adequate input voltage is found to balance the vertical buoyancy, the lateral force is sufficiently large to maneuver the bubble in the horizontal plane at high speed or, reversely, in the presence of flow conditions. By adjusting the input voltage, it is also possible to precisely position the bubble relative to boundaries introduced near the trapping volume (see *Methods* and *SI Appendix*, Fig. S3 for a discussion on the trap precision), thus overcoming the main limitation of standing-wave–based acoustical traps. An additional feature of the device is the ability to selectively trap a target bubble in crowded environments (Movie S3). This capacity is a result of the change in sign of the lateral force at approximately x=500 μm (Fig. 1*E*); bubbles beyond this location are repelled.

**Fig. 2.**
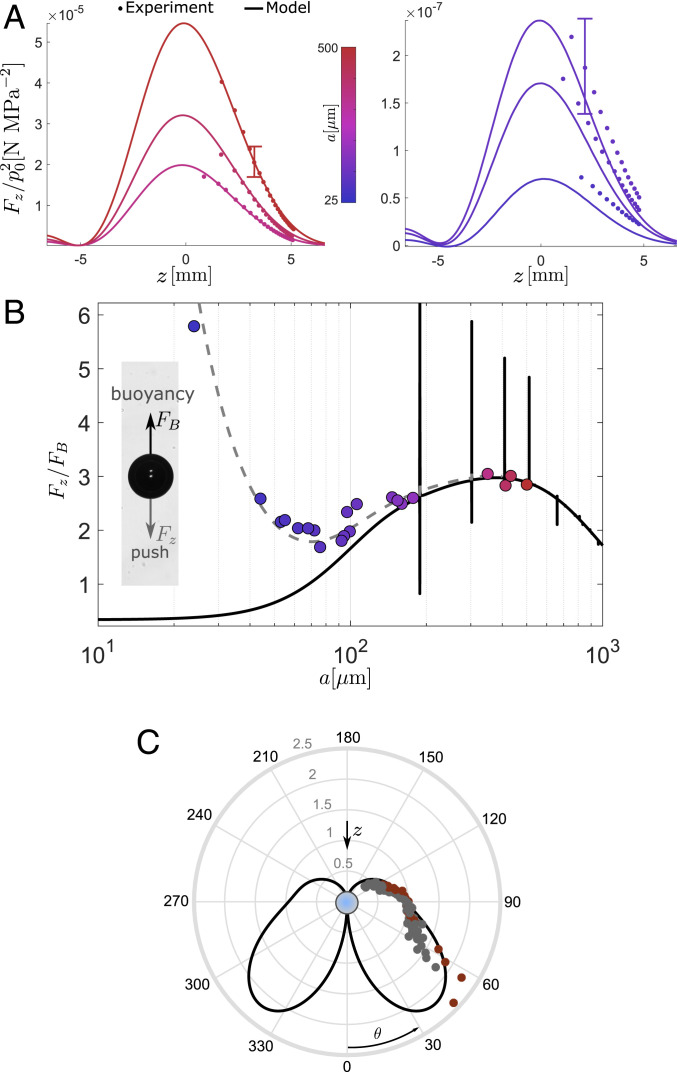
Pushing acoustic force and scattered field. (*A* and *B*) Force measurements by balancing the acoustic pushing force, $F_{z}$, and bubble buoyancy, $F_{B}$. (*A*) Measurements (solid circles) and model (solid lines) for bubbles in the range a=25 to 500 μm as a function of the distance to the trapping beam focus (z=0). (*B*) Interpolated values of the measured pushing force (solid circles) at z=4  mm, normalized by the bubble buoyancy, for $p_{0}=0.7\;$ MPa, compared to the acoustic force model alone (black solid line) and with the addition of a Stokes drag, $F_{s}=6\pi \mu av_{z}$, originating from an acoustic streaming flow ($v_{z}=8\;$ mm/s; see main text). (*C*) Direct hydrophone measure of the far-field scattering form function represented on a polar plot (see *SI Appendix* for more details). Model (solid lines) and measured (solid circles) diagrams for two independant bubbles are normalized with their respective value for $\theta =90^{○}$.

To test the accuracy of the force predictions, we performed experiments on n∼30 bubbles of varying size. The pushing force calibration relies on its precise balance with the upward buoyancy force (see *SI Appendix* for details on the experimental methods). For a sufficient driving power, $\left|F_{z}|=|F_{B}\right|$ is satisfied for two equilibrium positions, of which only one is stable, located downstream from the focus (z>0). Typical force measurements and error bars are shown in Fig. 2*A*. The solid lines are the predictions of the calculation and the solid circles are the experimental data points. We found a very good quantitative agreement for large bubbles for which the pushing force is large close to the beam focus and progressively decreases downstream (z>0). For smaller bubbles, the model systematically underestimates the total pushing force. Interpolating the experimental force measurements for all bubble sizes at a fixed driving pressure and at a fixed distance z=4.0 mm downstream from the focus revealed a transition for small bubbles of radii a<100 μm (Fig. 2*B*). The deviation from the prediction (Fig. 2*B*, solid black curve) originates from a net streaming flow, forced by the momentum flux transfer from the propagating beam to the viscous fluid bulk (27) (see *SI Appendix* for a detailed discussion on the effect of streaming and an evaluation of a critical bubble radius). Assuming a constant homogeneous flow with $u_{s}=8$ mm/s (for $p_{0}=0.7\;$ MPa) around the bubble, we can now add a net Stokes drag, $F_{s}=6\pi a\mu u_{s}$, where μ is the dynamic viscosity of water, and recover the experimental trend for smaller bubbles (Fig. 2*B*, dashed curve). For further details on the flow velocity estimation see *SI Appendix*, Fig. S4. As the bubble size decreases, we additionally anticipate a change in position of maximum pushing force and timescales for their manipulation (*SI Appendix*).

Next, we quantitatively investigated the absence of volume (monopolar) oscillations of the bubble positioned in the vortex core. Because the optical detection of the nanometric surface oscillations (∼50 nm for $p_{0}=1$ MPa and f=2.25 MHz) is challenging, we performed a direct acoustic measurement of the scattered pressure field, which carries information on the bubble oscillations. We exploited the good stability of the bubble in the trap to detect isolated bubble echos with a hydrophone placed at a distance, D=25 mm, much larger than the bubble radius and acoustic wavelength (far field). The polar diagram in Fig. 2*C* shows the far-field scattering amplitude as a function of the emission direction, θ (see *SI Appendix* for details on the measurement and *SI Appendix*, Fig. S5). The “wing-shaped” scattering diagram we observe experimentally indicates that monopolar bubble oscillations are largely reduced relative to higher-order modes and do not contribute to the scattered field. The data points closely follow the theoretical diagram which indicates very weak echo emissions as θ approaches π (*SI Appendix*, Fig. 5S*E*). The scattering diagram results from the superposition of other oscillation multipoles for which we can estimate the relative amplitude (*SI Appendix*, Fig. S5 *A*–*C*). For the two independent bubbles under consideration (a∼270 μm), dipolar, quadrupolar, and modes with order up to n=5 contribute to the oscillation and enhance the radiation toward the bubble rear. Remarkably, the suppression of the volume oscillation provides robustness to the trapping strategy: In strong pressure fields, volume oscillations are rapidly prone to nonlinear instabilities, which in turn destabilize the bubble’s position in standing wave traps (28). Both the force measurements and bubble echo detection confirm the linear nature of the scattering process involved in the trap.

### Trap Robustness through Thick Viscoelastic Layers.

**Fig. 3.**
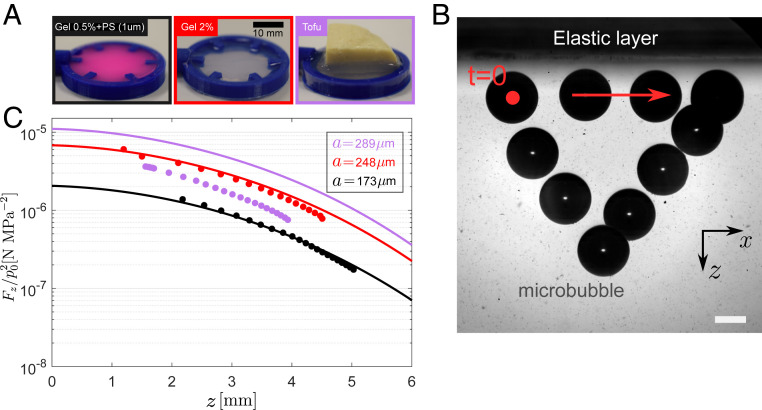
Trap robustness through opaque-to-light elastic layers. (*A*) Three different elastic layers were made of (*Left* to *Right*) a 0.5% agarose hydrogel with 1-μm fluorescent polystyrene beads, a 2% hydrogel, and a thick block of tofu. (Scale bar, 10 mm.) (*B*) Time-lapse photography of a bubble trapped and maneuvered with the trapping beam propagating through the elastic layer. (Scale bar, 100 μm.) (*C*) Force measurements with the elastic layers positioned on the propagation path between the trapping beam and the bubble.

A unique advantage of acoustic trapping over optical trapping is the ability to propagate through thick and optically opaque media. We characterized the robustness of the vortex wavefront topology to aberration and attenuation by introducing different viscoelastic layers in between the beam and the target bubble (Fig. 3*A*). We tested homogeneous agarose hydrogels with increasing stiffness (shear modulus G∼10 kPa), as well as a hydrogel containing dispersed 1-μm fluorescent polystyrene (PS) colloids, to combine acoustic scattering heterogeneity with optical opacity (see *Methods* for details on fabrication). We succeeded in trapping bubbles through all layers (Fig. 3*B*). As shown in Fig. 3*C*, when compared to the model, which does not include the presence of the viscoelastic layer, the measured force is in quantitative agreement for both the homogeneous and heterogeneous hydrogels, demonstrating a minimal effect on the trap. The main trapping characteristics, in both the lateral and axial directions, are also preserved upon propagation through an irregular, 2-cm-thick layer of tofu, whose properties accommodate for both the elastic properties and attenuation of typical biological tissue (29). The main feature is a measurable decrease in the trapping force consistent with a larger attenuation in tofu or reflection and refraction effects due to impedance mismatch at the two interfaces.

### Interaction of a Trapped Microbubble with a Viscoelastic Wall.

**Fig. 4.**
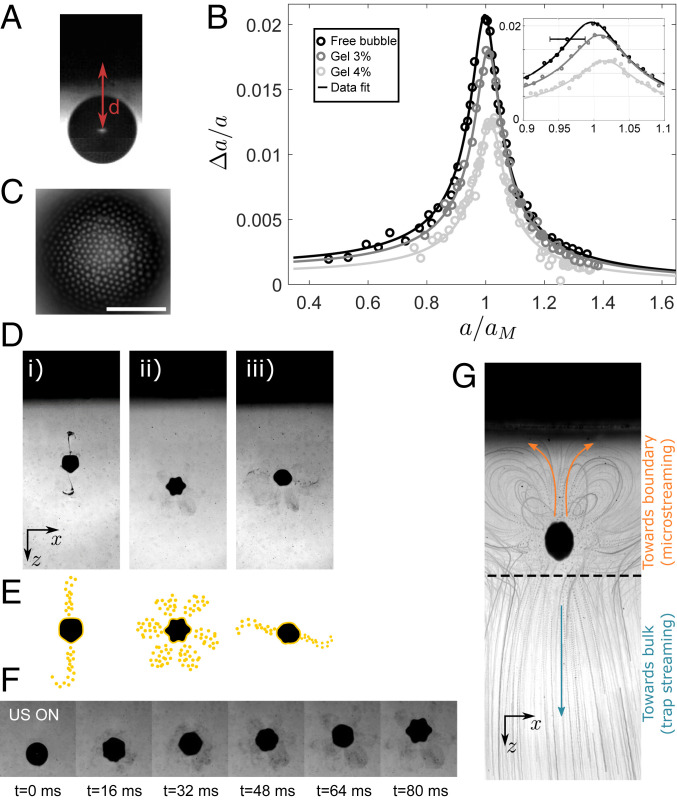
In situ bubble dynamics, payload release, and material transport guided by an acoustical trap. (*A*) Bubble positioned at a distance d=200±20 μm from a distant wall. (*B*) Driving bubble oscillations (Δa) with a secondary source of ultrasound (frequency $f^{\prime }=28.2\;$ kHz) as a function of bubble radius at rest, a. Shown are experimental points (circles) obtained for a “free bubble” far from any boundary (black) and a bubble at a distance d from a hydrogel wall with 3% (dark gray) and 4% (light gray) concentration of agarose. Black curves are the best fits to the data. In all curves, the bubble radius a is normalized by the theoretical Minnaert resonant size $a_{M}=115\;\mu$m. (*C–F*) Particle-coated bubbles as a model for in situ ultrasonic payload delivery. In the micrograph of a particle-coated bubble in *C*, the bubble interface is covered by 1 μm polystyrene particles. (Scale bar, 50 μm.) (*D* and *E*) Photographs and sketches of observed experimental particle release events. The bubbles are covered with 0.5 μm polystyrene particles. (*D*, *i*) Bidirectional release with particle plume toward the adjacent wall (surface mode n=5). (*D*, *ii*) Multidirectional release with six petal-like delivery sites (surface mode n=6). (*D*, *iii*) Bidirectional release with no particle plume toward the adjacent wall (surface mode in transition). (*F*) Experimental recording of the release event in *D*, *ii* during 2,000 acoustic cycles (Movie S5). (*G*) Subsequent transport of polystyrene particles toward the adjacent wall by bubble-generated microstreaming flows (*Upper* part of the photograph). Particles are also transported toward the bulk by the millimeter-scale flow generated by the trapping beam itself. (*D*, *F*, and *G*) Approximative bubble radius a=100 μm.

Neither bubble adhesion to the hydrogel nor the presence of a distant wall for detached bubbles prevented their manipulation. We thus exploited the good precision afforded by our trapping device to explore the influence of a distant wall on the dynamics of position-controlled microbubbles. Considerable interest exists in quantifying this effect, due to its importance in many situations involving microbubbles for contrast-enhanced imaging and therapy (30). However, few measurements have been possible due to the difficulty to simultaneously position, force, and measure the dynamics of isolated microbubbles (31). We integrated our trapping device with a high-speed imaging setup to achieve direct observation of acoustically forced bubble dynamics. Bubbles were positioned at a chosen distance, d, from the boundary and imaged from a side view (Fig. 4*A*). We forced larger, but slower, bubble oscillations with a secondary ultrasonic transducer ($f^{\prime }=28.2\;$ kHz) in combination with the trapping beam (f=2.25  MHz). See *SI Appendix* for details on the setup. We observed that this secondary source of ultrasound also generates a net acoustic force acting on the bubble, arising from the forced volume oscillations, Eq. **1**. However, in most cases it was still possible to operate the trapping beam to precisely position the bubble. Allowing for the slow dissolution of bubbles initially larger than their predicted resonant size ($a_{M}=115\;\mu$m for a monopolar volume oscillation at $f^{\prime }$), it was possible to observe the on- and off-resonance behavior for the same bubble, in both the linear and nonlinear regimes, with all other parameters fixed (Movie S4). Acquiring the bubble dynamics over a large range of bubble radii clearly revealed its resonant size with the micrometric resolution set by our optical setup (Fig. 4*B*). To our knowledge, despite its fundamental importance, the measurement of such resonance curves had remained impossible for a single bare microbubble, freely suspended in the liquid bulk away from any boundary. It also became possible to detect a shift in the bubble resonance size by introducing different distant walls made of agarose hydrogels with varying concentration. Although the shift is here modest (see *SI Appendix* for details), its quantification can have important implications for targeted contrast agent imaging protocols, where it is important to differentiate the dynamics of bound, unbound but adjacent, and freely circulating microbubbles and relate them to the mechanical properties of tissue (21).

### Directed Payload Delivery from Microbubble Cargos.

Finally, the manipulation principle we introduced can be applied to nanoparticle-coated microbubbles (Fig. 4*C*), which we used as surrogates to drug-loaded microbubbles, which are currently being considered to achieve local delivery of therapeutic agents to target tissue (21, 32). Recent reports have pinpointed the importance of nonspherical bubble oscillations and microstreaming in the release and transport mechanisms of nanopoarticles (33–36) but, in all of these studies, the microbubbles were either pinned to a rigid substrate (33–35) or allowed to rise against cell monolayers until contact (36). Both situations may have important implications for the observed delivery mechanisms.

We maneuvered nanoparticle-coated microbubbles to a fixed distance from a hydrogel boundary before activating the secondary source of ultrasound. For coated bubbles close to their resonant size (a∼120 μm), large-amplitude nonspherical oscillations can lead to an efficient release in one or several directions, over a distance greater than a few bubble diameters (Fig. 4 *D*–*F* and Movie S5). Interestingly, the generation of particle “plumes” oriented toward the physical boundary was frequently observed (e.g., Fig. 4 *D*, *i*). These plumes result from a violent particle release process ejecting particles with terminal speeds three to five times larger than the typical microstreaming flow velocities we observed around the oscillating bubble. Plume formation seems to be favored by the initially high surface coverage, easily identified for bubbles exhibiting gravity-driven desorption before acoustic activation (37) (Movie S5). The plumes frequently appeared at locations of maximal acceleration of the bubble interface (38) undergoing nonlinear parametric shape oscillations with typical mode orders, n=4,5,6, for the bubble sizes considered. Mode transitions from high to lower orders during the bubble surface oscillation were also frequently observed to trigger ejection plumes. A definitive understanding of this plume formation mechanism will require the integration of a setup such as the one presented here with high magnification and high-speed imaging, to detect real-time changes in the bubble’s surface microstructure.

## Discussion

We have demonstrated the use of a vortex beam-based acoustical trap to manipulate and activate individual microbubbles in a complex environment including viscoelastic walls. Several key differences distinguish this trap from optical manipulation (31, 39, 40) or standing acoustic wave traps also introduced for microbubbles (14). The vortex trapping beam can operate after propagating through thick, opaque-to-light, viscoelastic media without suffering from wavefront distortion. It can be sharply focused to localized sites, offering a pinpoint control over an individual microbubble, even in crowded environments. The operation frequency used is standard in many ultrasonic applications including diagnostic and therapeutic ultrasound. Because the trap relies on linear (gentle) center-of-mass translations and surface oscillations, it does not suffer from the onset of nonlinear and unstable volume oscillations at high acoustic pressures. Acoustic traps based on other beam configurations (41–43) may be relevant in the present context.

Here we manipulated microbubbles that were typically one to two orders of magnitude larger than conventional, clinically approved, microbubble-based contrast agents (21). Using a higher frequency for the trap should enable a better spatial resolution and improved trap stiffness on micrometer-sized bubbles (see extended discussion in *SI Appendix*). Nevertheless, viscous dissipation of the vortex beam occurs in the liquid bulk, leading to the typical streaming flow we observed here, but may also appear in the boundary layer immediately out of the bubble’s interface (44). A precise quantification of the magnitude of each of these dissipation effects would represent an interesting perspective to this work, particularly for its use to control clinically approved contrast agents. The manipulation experiments we presented make acoustical traps a viable tool to perform position-controlled in vitro studies with microbubbles that could result in being particularly useful to validate the predictive power of hydrodynamic models accommodating the presence of the bubble’s complex environment.

The all-acoustical strategy to manipulate and activate microbubbles we presented could be combined with single-bubble detection (23) and paves the way to develop and control biomedical operations such as energy focusing at the microscale, local cellular deformation and rupture with shear stresses, and the controlled and directed release of therapeutic agents, with potential in vivo applications (45).

## Methods

### Experimental Setup.

The setup is shown in *SI Appendix*, Fig. S2 and consists of an open-top tank (200 × 250 × 500 mm) filled with tap water and partially lined with an acoustic absorber (Aptflex F28; Precision Acoustics Ltd.) to avoid reflection toward the trapping region. No particular care was taken to degas or purify the propagation medium. Given the relative high frequency (f=2.25  MHz) and moderate acoustic intensities generated (<50 W/${cm}^{2}$), no onset of cavitation was observed (mechanical index [MI] < 1). The room temperature was set to 21 °C with a typical fluctuation of ±1 °C. Vortex beams can be generated with a minimum of four ultrasonic transducers (46). Here the trapping beam was generated by a spherical (focused) ultrasonic transducer divided into eight azimuthal piezoelectric sectors (Imasonics Ltd.). The geometrical focus that determines the working distance is F=38  mm for a transducer diameter D=40  mm. An in-house electronic board is used to delay the driving signals by 1/(8f)∼55  ns of each sector relative to its immediate neighbor. This results in an acoustic phase singularity on the propagation axis and the helicoidal structure of the vortex-field wavefronts (topological charge m=1).

The driving signal feeding the electronic board was generated by a waveform generator (33220A; Agilent) and amplified with a 100-W radio-frequency amplifier (AG1021; T&C Power Conversion Inc.). For the acoustic-field measurements, bursts of N=10 sinusoidal cycles are used at low driving powers. The acoustic field is scanned with a calibrated 75-μm needle hydrophone (Precision Acoustics Ltd.) in combination with a signal booster amplifier and a digital oscilloscope. For trapping experiments, the number of cycles is increased to N=200 with a burst repetition period of 200 μs resulting in a 44% duty cycle. Typically, the peak pressure on the acoustic vortex ring, $p_{0}$, was set between 0.35 and 1.15 MPa to maneuver bubbles in the 20- to 500-μm range.

To image the trapping zone, we used a high-working distance adjustable magnification lens (6.5× zoom lens; Thorlabs). This allows us to image through the Plexiglas wall of the tank with a magnification up to 10× at a working distance of approximately 90  mm. Either a compact scientific camera (DCC1545M; Thorlabs) was used for slow manipulation of the bubble or a high-speed camera (Fastcam SA5; Photron) was used for streaming flow observation (≈1,000 frames per second) or bubble dynamics (≈300,000 fps). An optical fiber light is placed in a protection sleeve and directly immersed in the water tank at approximately 60  mm from the optical imaging plane. To initially align the optical imaging plane with the acoustical trapping zone, we scanned the acoustic field with the needle hydrophone and placed the tip laterally in the acoustical vortex core and axially at the transducer’s geometrical focus. This step is also used to precisely calibrate the image pixel size from the previously characterized size of the needle hydrophone with a 20× microscope objective. The resolution could be adjusted down to 2.5 μm using the high-speed camera sensor. The bubbles were trapped downstream from the acoustic focus in the optical imaging plane.

### Microbubble Preparation.

Bare bubbles were generated with an in-house electrolysis setup that consists of a pair of copper wires (80 μm in diameter) connected to a 9-V battery. The wires were mounted in a waterproof casing. Due to the high conductivity of the nonpurified water, the cathode could be placed arbitrarily in the tank. The anode was positioned near the trapping zone with the 3D translation stage. The electrolysis generated small oxygen bubbles (∼40 μm diameter) at the anode. Larger bubbles could be obtained from successive coalescence of smaller bubbles.

Particle-coated bubbles were made using charge-stabilized polystyrene particles (ThermoFisher Scientific, Molecular Probes) of 500 nm diameter. To promote adsorption to the water–air interface, the particles were suspended in an aqueous solution of 500 mM NaCl (VWR Chemicals; AnalaR NORMAPUR, 99.5%). Ultrapure water with resistivity 18.2 MΩ cm (Milli-Q system; Millipore) was used to prepare the initial solution. The mechanical agitation of a 0.4% wt/vol particle suspension using a vortex mixer would typically result in the generation of polydisperse (10 to 400 μm) particle-coated bubbles. The bubbles were gently collected by aspiration with a pipette and injected in the water tank where they were allowed to rise up to a hydrogel membrane mounted on the 3D translation stage.

### Hydrogel and Tofu Preparation.

Agarose gels with varying viscoelastic properties were obtained by tuning the gel concentration. We used four concentrations, 1, 2, 3, and 5% wt/vol. Agarose powder (A9539; Sigma Aldrich) is mixed with ultrapure water (resistivity 18.2 MΩ cm; Milli-Q filtration system, Millipore) at room temperature. The solution is heated to 145 °C and stirred for 10 min until the solution becomes transparent, then poured into a sample container, and allowed to set at room temperature to form a layer with a thickness of 8 mm. Visible bubbles were quickly removed from the setting gel. The sample holder was 3D printed and positioned on a clean glass slide to obtain a smooth gel boundary. The tofu sample was prepared by cutting an irregular piece of dimensions ∼20 × 20 × 20 mm out of a commercially available block of tofu (The Tofoo Co.; Naked tofu). It was positioned simultaneously in the sample container with a 1% agarose setting hydrogel.

### Data Availability.

The datasets generated and analyzed during this study are available in the OSF repository: https://osf.io/jy9qz/.

## Supplementary Material

Supplementary File

Supplementary File

Supplementary File

Supplementary File

Supplementary File

Supplementary File

## References

[1] A. Ashkin, J. M. Dziedzic, J. E. Bjorkholm, S. Chu, Observation of a single-beam gradient force optical trap for dielectric particles. Opt. Lett. 11, 288–290 (1986). 19730608 10.1364/ol.11.000288

[2] R. Pethig, Dielectrophoresis: Status of the theory, technology, and applications. Biomicrofluidics 4, 022811 (2010). 20697589 10.1063/1.3456626 PMC2917862

[3] C. Gosse, V. Croquette, Magnetic tweezers: Micromanipulation and force measurement at the molecular level. Biophys. J. 82, 3314–3329 (2002). 12023254 10.1016/S0006-3495(02)75672-5 PMC1302119

[4] K. Svoboda, S. M. Block, Biological applications of optical forces. Annu. Rev. Biophys. Biomol. Struct. 23, 247–285 (1994). 7919782 10.1146/annurev.bb.23.060194.001335

[5] K. C. Neuman, A. Nagy, Single-molecule force spectroscopy: Optical tweezers, magnetic tweezers and atomic force microscopy. Nat. Methods 5, 491–505 (2008). 18511917 10.1038/nmeth.1218 PMC3397402

[6] T. Laurell, F. Petersson, A. Nilsson, Chip integrated strategies for acoustic separation and manipulation of cells and particles. Chem. Soc. Rev. 36, 492–506 (2007). 17325788 10.1039/b601326k

[7] G. Sitters, Acoustic force spectroscopy. Nat. Methods 12, 47–50 (2015). 25419961 10.1038/nmeth.3183

[8] J. P. K. Armstrong, Engineering anisotropic muscle tissue using acoustic cell patterning. Adv. Mater. 30, 1802649 (2018). 10.1002/adma.201802649 PMC6386124 30277617

[9] Z. Ma, Acoustic holographic cell patterning in a biocompatible hydrogel. Adv. Mater. 32, e1904181 (2019). 31782570 10.1002/adma.201904181

[10] J.-L. Thomas, R. Marchiano, D. Baresch, Acoustical and optical radiation pressure and the development of single beam acoustical tweezers. J. Quant. Spectrosc. Radiat. Transf. 195, 55–65 (2017).

[11] A. Ozcelik, Acoustic tweezers for the life sciences. Nat. Methods 15, 1021–1028 (2018). 30478321 10.1038/s41592-018-0222-9 PMC6314293

[12] D. Baresch, J.-L. Thomas, R. Marchiano, Observation of a single-beam gradient force acoustical trap for elastic particles: Acoustical tweezers. Phys. Rev. Lett. 116, 024301 (2016). 26824541 10.1103/PhysRevLett.116.024301

[13] X. Ding, On-chip manipulation of single microparticles, cells, and organisms using surface acoustic waves. Proc. Natl. Acad. Sci. U.S.A. 109, 11105–11109 (2012). 22733731 10.1073/pnas.1209288109 PMC3396524

[14] A. Eller, Force on a bubble in a standing acoustic wave. J. Acoust. Soc. Am. 43, 170–171 (1968).

[15] D. Foresti, M. Nabavi, M. Klingauf, A. Ferrari, D. Poulikakos, Acoustophoretic contactless transport and handling of matter in air. Proc. Natl. Acad. Sci. U.S.A. 110, 12549–12554 (2013). 23858454 10.1073/pnas.1301860110 PMC3732964

[16] M. Baudoin, Folding a focalized acoustical vortex on a flat holographic transducer: Miniaturized selective acoustical tweezers. Sci Adv. 5, eaav1967 (2019). 30993201 10.1126/sciadv.aav1967 PMC6461452

[17] A. Marzo, B. W. Drinkwater, Holographic acoustic tweezers. Proc. Natl. Acad. Sci. U.S.A. 116, 84–89 (2019). 30559177 10.1073/pnas.1813047115 PMC6320506

[18] L. Andrique, A model of guided cell self-organization for rapid and spontaneous formation of functional vessels. Sci. Adv. 5, eaau6562 (2019). 31206014 10.1126/sciadv.aau6562 PMC6561743

[19] K. Hynynen, N. McDannold, N. Vykhodtseva, F. A. Jolesz, Noninvasive MR imaging–guided focal opening of the blood-brain barrier in rabbits. Radiology 220, 640–646 (2001). 11526261 10.1148/radiol.2202001804

[20] P. Marmottant, S. Hilgenfeldt, Controlled vesicle deformation and lysis by single oscillating bubbles. Nature 423, 153–156 (2003). 12736680 10.1038/nature01613

[21] E. C. Unger, Therapeutic applications of lipid-coated microbubbles. Adv. Drug Deliv. Rev. 56, 1291–1314 (2004). 15109770 10.1016/j.addr.2003.12.006

[22] G. Tsivgoulis, Safety and efficacy of ultrasound-enhanced thrombolysis: A comprehensive review and meta-analysis of randomized and nonrandomized studies. Stroke 41, 280–287 (2010). 20044531 10.1161/STROKEAHA.109.563304

[23] C. Errico, Ultrafast ultrasound localization microscopy for deep super-resolution vascular imaging. Nature 527, 499–502 (2015). 26607546 10.1038/nature16066

[24] T. J. Asaki, P. L. Marston, Acoustic radiation force on a bubble driven above resonance. J. Acoust. Soc. Am. 96, 3096–3099 (1994).

[25] M. S. Plesset, A. Prosperetti, Bubble dynamics and cavitation. Annu. Rev. Fluid Mech. 9, 145–185 (1977).

[26] D. Baresch, J.-L. Thomas, R. Marchiano, Three-dimensional acoustic radiation force on an arbitrarily located elastic sphere. J. Acoust. Soc. Am. 133, 25–36 (2013). 23297880 10.1121/1.4770256

[27] C. Eckart, Vortices and streams caused by sound waves. Phys. Rev. 73, 68 (1948).

[28] P. Koch, T. Kurz, U. Parlitz, W. Lauterborn, Bubble dynamics in a standing sound field: The bubble habitat. J. Acoust. Soc. Am. 130, 3370–3378 (2011). 22088010 10.1121/1.3626159

[29] J. Wu, Tofu as a tissue-mimicking material. Ultrasound Med. Biol. 27, 1297–1300 (2001). 11597372 10.1016/s0301-5629(01)00424-0

[30] M. Overvelde, Dynamics of coated microbubbles adherent to a wall. Ultrasound Med. Biol. 37, 1500–1508 (2011). 21816289 10.1016/j.ultrasmedbio.2011.05.025

[31] V. Garbin, Changes in microbubble dynamics near a boundary revealed by combined optical micromanipulation and high-speed imaging. Appl. Phys. Lett. 90, 114103 (2007).

[32] I. Lentacker, S. C. De Smedt, N. N. Sanders, Drug loaded microbubble design for ultrasound triggered delivery. Soft Matter 5, 2161–2170 (2009).

[33] Y. Luan, Lipid shedding from single oscillating microbubbles. Ultrasound Med. Biol. 40, 1834–1846 (2014). 24798388 10.1016/j.ultrasmedbio.2014.02.031

[34] V. Poulichet, V. Garbin, Ultrafast desorption of colloidal particles from fluid interfaces. Proc. Natl. Acad. Sci. U.S.A. 112, 5932–5937 (2015). 25922529 10.1073/pnas.1504776112 PMC4434748

[35] G. Lajoinie, Non-spherical oscillations drive the ultrasound-mediated release from targeted microbubbles. Commun. Phys. 1, 22 (2018).

[36] R. Silke, Sonoprinting of nanoparticle-loaded microbubbles: Unraveling the multi-timescale mechanism.Biomaterials 217, 119250 (2019). 31288172 10.1016/j.biomaterials.2019.119250

[37] J. W. Tavacoli, G. Katgert, E. G. Kim, M. E. Cates, P. S. Clegg, Size limit for particle-stabilized emulsion droplets under gravity. Phys. Rev. Lett. 108, 268306 (2012). 23005023 10.1103/PhysRevLett.108.268306

[38] V. Poulichet, A. Huerre, V. Garbin, Shape oscillations of particle-coated bubbles and directional particle expulsion. Soft Matter 13, 125–133 (2017). 10.1039/c6sm01603k PMC5304335 27714376

[39] B. T. Unger, P. L Marston, Shape oscillations of particle-coated bubbles and directional particle expulsion. J. Acoust. Soc. Am. 83, 970 (1988).

[40] P. Prentice, A. Cuschieri, K. Dholakia, M. Prausnitz, P. Campbell, Membrane disruption by optically controlled microbubble cavitation. Nat. Phys. 1, 107 (2005).

[41] D. Baresch, J.-L. Thomas, R. Marchiano, Spherical vortex beams of high radial degree for enhanced single-beam tweezers. J. Appl. Phys. 113, 184901 (2013).

[42] P. Zhang, Generation of acoustic self-bending and bottle beams by phase engineering. Nat. Commun. 5, 1–9 (2014). 10.1038/ncomms5316 24989825

[43] A. Franklin, A. Marzo, R. Malkin, B. W. Drinkwater, Three-dimensional ultrasonic trapping of micro-particles in water with a simple and compact two-element transducer. Appl. Phys. Lett. 111, 094101 (2017).

[44] D. Baresch, J.-L Thomas, R. Marchiano, Orbital angular momentum transfer to stably trapped elastic particles in acoustical vortex beams. Phys. Rev. Lett. 121, 074301 (2018). 30169074 10.1103/PhysRevLett.121.074301

[45] P. Dayton, A. Klibanov, G. Brandenburger, K. Ferrara, Acoustic radiation force in vivo: A mechanism to assist targeting of microbubbles. Ultrasound Med. Biol. 25, 1195–1201 (1999). 10576262 10.1016/s0301-5629(99)00062-9

[46] B. T. Hefner, P. L. Marston, An acoustical helicoidal wave transducer with applications for the alignment of ultrasonic and underwater systems. J. Acoust. Soc. Am. 106, 3313–3316 (1999).

