# Supplementary material for: Acoustic trapping of microbubbles in complex environments and controlled payload release
Source: Proc Natl Acad Sci U S A. 2020 Jun 22;117(27):15490–6. doi: 10.1073/pnas.2003569117 (PMC7354944; doi:10.1073/pnas.2003569117)
Supplement: Supplementary File [file pnas.2003569117.sapp.pdf]

Acoustic trapping of microbubbles in complex environments and controlled payload release

Diego Baresch[1,2] and Valeria Garbin[1,3]

Department of Chemical Engineering, Imperial College London, London SW7 2AZ, UK

University of Bordeaux, CNRS, I2M, UMR 5295, F-33405 Talence, France

Department of Chemical Engineering, Delft University of Technology, Delft, The Netherlands

Supporting Information

1. Analytical model for the bubble trap

Incident vortex beam, scattered field and acoustic force To calculate the 3D acoustic force exerted on the bubble arbitrarily positioned in the ultrasonic field we used the method developed in Ref. (1). In brief, the monochromatic incident beam is expanded in a set of spherical harmonics in the spherical basis centered on the bubble (r, θ, φ) as:

$$\phi = \phi_0 e^{-i\omega t} \sum_{n=0}^{\infty} \sum_{m=-n}^{n} A_n^m j_n(kr) Y_n^m(\theta, \varphi), \quad [1]$$

where ϕ is the complex acoustic velocity potential related to the real acoustic pressure change as $p = -\rho \Re\left(\frac{\partial \phi}{\partial t}\right)$. $\rho = 1000$ kg/m^3 is the fluid density, j_n are spherical Bessel functions of the first kind, $Y_n^m = P_n^m(\cos\theta) e^{im\varphi}$ are complex spherical harmonics that involve associated Legendre polynomials, P_n^m, $\omega = 2\pi f$ is the angular frequency, $k = \omega/c$ is the wavenumber in the liquid medium and $c = 1485$ m/s the speed of sound at 20° Celsius in water. The beam shape coefficients A_n^m describe the incident ultrasonic field in the spherical basis and can be accurately calculated for focused vortex fields for a specified transducer geometry (2). The theoretical pressure field is shown in Fig.1A (modulus and phase) and compared to hydrophone measurements in Fig.1 B.

The scattered field is expanded in the same basis, leading to a potential in the far-field ($r \gg \lambda$) written as

$$\phi_s = \phi_0 \frac{a}{r} e^{i(kr - \omega t)} f_s(\theta, \varphi), \quad [2]$$

$$f_s(\theta, \varphi) = \frac{1}{ka} \sum_{n=0}^{\infty} \sum_{|m|<n} i^{-n-1} R_n A_n^m Y_n^m(\theta, \varphi). \quad [3]$$

$f_s(\theta, \varphi)$ is the complex scattering form function. It was used to plot the polar diagrams in Fig.2C and Supplementary Figure 5. The scattering coefficients R_n for a bubble are those of a fluid spherical object (see *e.g.* Ref.(3), where n is the order of the oscillating multipole ($n = 0$ for monopolar, $n = 1$ for dipolar, $n = 2$ quadrupolar and so on)

In a final step, following the method described in Ref.(1), the force (nonlinear) is calculated from the total field (linear) for any location of the bubble relative to the incident vortex beam. This assumes that the bubble undergoes linear oscillations, which has been confirmed experimentally by the good agreement in between the model and force measurements (Figure 2A), but also from the detected bubble echos (Supplementary Fig.5). A plot of the vertical and lateral forces are shown in Figure 1D and E, as functions of the vertical (z) and lateral (x) displacements respectively. The vertical force, F_z, is also plotted as a function of the bubble radius, a, in Fig.2 B.

Force on a small bubble The mechanisms underpinning the acoustic force and the relevant physical quantities involved are difficult to identify from the modal decomposition presented so far. In the following, we will restrict the discussion to small bubbles relative to the driving wavelength, $a/\lambda \ll 1$ (long wavelength limit), for which an explicit expression for the nonlinear force can be given (4):

$$\vec{F} = -\frac{1}{2}\left\{\Re(\alpha_m)\frac{1}{2\rho c^2}\vec{\nabla}|p|^2 - \Re(\alpha_d)\frac{1}{2}\rho\vec{\nabla}|\vec{v}|^2\right\}, \quad [4]$$

where p and $\vec{v}$ are the first order acoustic pressure and velocity fields taken at the bubble center respectively. $\Re$ denotes the real part of complex quantities. α_m and α_d are two acoustic strength parameters associated to the monopolar and dipolar oscillation modes of a spherical particle. Eq.[4] is restricted to the "gradient" force exerted on a bubble. This component dominates the "scattering" force for configurations involving focused beams (with non-zero energy gradients) (4). The strength parameters can be determined from a Taylor expansion of the scattering coefficients R_0 and R_1 respectively (Eq.[3]), taking $a/\lambda \ll 1$. For the special case of a bubble, their real part can be expressed as (5):

$$\Re(\alpha_m) = \frac{4}{3}\pi a^3 \frac{K}{K_b}\frac{\omega_0^2(\omega^2-\omega_0^2)}{(ka\omega^2)^2+(\omega^2-\omega_0^2)^2} \quad [5]$$

$$\Re(\alpha_d) = 4\pi a^3\left(\frac{\rho_b-\rho}{2\rho_b+\rho}\right) \sim -4\pi a^3. \quad [6]$$

$K=\rho c^2$ and $K_b=\rho_b c_b^2$ are the propagation medium and gas bulk modulii respectively, ρ_b the gas density and $\omega_0=(3K_b/\rho a^2)^{1/2}$ the natural oscillation frequency of the bubble (or Minnaert resonance frequency). This frequency neglects thermal dissipation in the bubble and surface tension. Similarly, the expression for the strength parameters α_m and α_d has been obtained in the case of an inviscid, non-heat conducting, external liquid. Corrections have recently been given using a similar formalism, making them easy to implement in this derivation (6). Nevertheless, such corrections will be discarded for the purpose of the present discussion.

The bulk modulus of the gas is much weaker compared to that of the fluid such that $\Re(\alpha_m)/\Re(\alpha_d) \sim K/K_b \sim 10^3$ for bubbles off-resonance. Therefore, in the long wavelength regime, the acoustic force is generally and correctly assumed to be a result of monopolar volume oscillations of the bubble alone (first term in Eq.[4]). For instance, using Eq.[5] in Eq.[4], we have retrieved the expression for the primary Bjerknes force as derived by Crum and Prosperetti when thermo-viscous damping effects are neglected (7). A bubble located off-axis of the vortex beam will undergo monopolar oscillations, and will similarly be driven by a force proportional to $|\vec{\nabla}p|$ towards, or away from, the propagation axis, for bubbles driven above or bellow resonance respectively. When the bubble reaches the propagation axis, z, vortex beams create the specific condition for which $|p|=0$ and $\frac{\partial|p|^2}{\partial z}=0$ simultaneously at $r=\sqrt{x^2+y^2}=0$, which could be termed a nodal line, or screw dislocation line (8). Under these conditions, the axial force exerted by a vortex beam written as $p=\tilde{p}(r,z)e^{i(m\theta-\omega t)}$, where m quantifies the beam helicity, reads

$$F_z = \frac{\Re(\alpha_d)}{4\rho\omega^2}\frac{\partial}{\partial z}\left|\frac{\partial\tilde{p}}{\partial r}\right|^2, \quad [7]$$

for a bubble (or any other spherical object) located on-axis ($r=0$). The pushing force will be much weaker than the lateral trapping force (by approximately 3 orders of magnitude). It's magnitude will strongly depend on the capacity of the trapping device to maximize both the lateral and axial gradients of the acoustic pressure field. The expression of $\Re(\alpha_d)$ also indicates that the pushing force is largely insensitive to the density of the gas. It scales with the bubble volume a^3, making the trapping vertical position against

buoyancy unaffected by changes of the bubble size. This remains true as long as the effect of acoustic streaming flows can be neglected (see discussion bellow).

It is now convenient to express the axial force acting on a bubble using a model for the incident beam used in our experiments. Laguerre-Gaussian (LG) modes are a useful description of vortex beams in the paraxial approximation (*i.e.* weak focusing) (9, 10). Setting the azimuthal and radial index of the LG mode to respectively 1 and 0, the pressure field can be modelled as:

$$\tilde{p}(r,z) = P_{LG}\left(\frac{rw_0}{w(z)^2}\right)\exp\left(-\frac{kr^2z}{2(z^2+z_R^2)}\right)\exp\left(i[kz+g(z)]\right) \qquad [8]$$

where $P_{LG} = p_0\sqrt{2}\exp[1/2]$ is a constant proportional to the maximum pressure measured on the vortex ring at focus, p_0. $w_z = w_0(1+(z/z_R)^2)$ and w_0 are the beam waist and beam waist at focus ($z=0$) respectively while $z_R = kw_0^2/2$ is the Rayleigh distance. $g(z)$ describes the phase shift of π as the wave passes the focus, also known as the Gouy phase. Using Eq.[8] in Eq.[7] gives the pushing force acting on the bubble:

$$F_{LG} = -\frac{P_{LG}^2}{\rho\omega^2}\frac{z}{z_R^2}\frac{w_0^2}{w(z)^6}\Re(\alpha_d) \sim 4\pi a^3\frac{P_{LG}^2}{\rho\omega^2}\frac{z}{z_R^2}\frac{w_0^2}{w(z)^6}. \qquad [9]$$

The theoretical LG mode and the experimental lateral pressure scan at the focal plane are compared in Supplementary Figure 1A. The agreement is good near the vortex core (with the beam waist chosen as $w_0 = 1.025\lambda$ (11)), but the LG model cannot capture effects of diffraction (*e.g. secondary lobes*) seen in experiment or with a spherical decomposition of the beam (see Eq.[1] and Figure 1D in the main text), as a consequence of the paraxial approximation. This is also confirmed by looking at the force profile shown in Supplementary Figure 1B. While the correct magnitude of the pushing force is predicted by Eq.[9] (for $a = 5$ μm and $p_0 = 1$ MPa), it shows a different evolution with z. In particular it predicts a stiffer axial trap than that observed with the full analytical model (1, 2). Nevertheless, the LG model is useful in predicting the maximum pushing force, F_{LG}, found downstream from the focus at $z_{max} = z_R/\sqrt{5}$ and reads

$$F_{LG} = C\frac{a^3p_0^2}{\rho\omega^2}\left(\frac{\lambda}{w_0^4}\right). \qquad [10]$$

Here $C = 4\exp[1]\sqrt{5}^5/108 \sim 6$ is a calculation constant. This equation indicates that to retain a maximum pushing force, it is necessary to minimize the beam waist, w_0, chosen as close as possible to λ, the theoretical limit being $w_0 \sim \lambda F/D \sim \lambda/2$ where F and D are respectively the focal length and aperture diameter of the spherical transducer.

It is important to additionally note that the position of maximum pushing force is size dependent. It will progressively shift from $z=0$ for large bubbles, in a regime dominated by the "scattering" component of the force (Fig.2 A), to $z \sim z_R$, for small bubbles, in a regime dominated by the "gradient" component of the force (Supp. Fig.1 B).

Viscous drag due to acoustic streaming The ultrasonic beam that propagates in the fluid is affected by thermo-viscous absorption. Therefore, a small amount of momentum is transferred to the bulk leading to the rise of a streaming flow, $\vec{v}_s$, which is also a nonlinear quantity induced by the propagating linear field (12). The exact modeling of this flow is usually mathematically involved and highly dependant on the ascribed hydrodynamic boundary conditions. Nevertheless, it will be quadratic with the driving acoustic pressure and its component along z will be written:

$$u_s = p_0^2\chi_s \qquad [11]$$

where χ_s will be a function mainly determined by the acoustic frequency , medium attenuation, properties of the acoustic beam and, importantly, of the hydrodynamic boundary conditions. From the qualitative measurements presented in the following section (see Supplementary Figure 4B), the acoustic streaming flow has been estimated to be $u_s \sim 4.5$ mm/s for $p_0 = 0.7$ MPa and a duty cycle of 44%.

Our measurements also indicate that u_s varies slowly with the lateral, x, and axial, z, distance from the focus, and can thus be assumed to be constant for $0 < z < 2$ mm. In that region, the bubble will be affected by a viscous drag:

$$F_s = Du_s, \quad [12]$$

where we have assumed the drag coefficient to be $D = 6\pi\mu a$, for bubbles with presumably contaminated surfaces and where $\mu \sim 10^{-3}$ Pa·s is the viscosity of water.

Critical bubble radius and trap behaviour Given the timescale we observed for bubble acceleration in our force balance experiments, $\tau \sim 1$ ms, we can safely neglect inertial effects (bubble acceleration, added-mass and Basset forces) and the bubble will obey a simple balance:

$$F_B = F_z + 6\pi\mu a(u_s - u_b) \quad [13]$$

in between the bubble buoyancy, acoustic pushing force and viscous drag respectively, where u_b is the bubble velocity which is equal to zero at equilibrium. The three forces are plotted in Supplementary Figure 1C, for $p_0 = 0.5$ MPa and $z = z_{max}$. As previously discussed, the buoyancy and acoustic force both scale with the bubble volume, a^3. However for small bubbles, the drag force arising from acoustic streaming progressively dominates the force balance as it decreases slower with a.

The critical radius a_c for which the acoustic force is equal to the viscous drag is found to be around 60 μm (in agreement with our experimental results, Fig.2B in the main text). For bubbles of size greater than a_c, the trap dynamics will be dictated by the establishment of the acoustic force, over a few acoustic cycles (microseconds), and can be considered instantaneous in Eq.[13]. The buoyancy can be simply and rapidly balanced by adjusting the driving pressure, p_0, and the equilibrium position will remain stable with changes in bubble size. However, a_c is found to be close to the biggest radius admissible for the long wavelength regime to hold, and, rapidly, the trapping position will depend on the bubble size again (Fig. 2B). For bubble sizes well bellow the critical radius, the balance will be dominated by the streaming flow which is generated in a much slower timescale. The build-up of the streaming flow will depend on various parameters including the fluid viscosity, the rate of absorption of the incident beam and boundary conditions. Our observations indicate a much slower timescale, tens to hundreds of milliseconds. The trap behaviour in this regime can become difficult to adjust for rapidly shrinking bubbles. Mitigating the viscous drag to facilitate the trapping of micron size bubbles will ultimately depend on the possibility to reduce χ_s in Eq.[11] by changing the driving frequency, beam waist or boundary conditions. A definitive answer requires further investigation and numerical modelling.

2. Experimental characterization of the bubble trap

Acoustic force At each equilibrium position, the acoustic pushing force F_z is equal in magnitude to the buoyancy, F_B. For varying driving amplitudes p_0, we measure both the bubble radius a and position, z relative to the acoustic focus. By computing the quantity $Q = F_B/p_0^2$, we can compare our measurements to the corresponding theoretical curve. Because of the 44% emission duty cycle we used, the theoretical force curves must be reduced by the same amount in order to be compared with the experiments. Our experimental uncertainty, δQ, is essentially due to the precision in measuring a, for which $\delta a/a$ is typically in between 1% and 3% for big ($a \sim 300$ μm) and small ($a \sim 100$ μm) bubbles respectively and to the systematic pressure measurement error introduced by our hydrophone, $\delta p_0/p_0 = 10\%$. This gives a total error $\delta Q = 3\delta a/a + 2\delta p_0/p_0$ lying in between 20% and 30% for the range of bubbles we studied. Though the systematic error seems large, reversely, by performing a statistical analysis on the force measurement curves on a large number of big bubbles constitutes a possibility to calibrate the pressure field of a transducer with a reduced error. For $n = 8$ bubbles ($a > 150$ μm), the best fit of the experimental force curves gives the amplitude of the drive pressure p_0 that we used to plot the theoretical curves in Figure 2 A-B and E.

The vertical positioning precision and bubble and relative fluctuations were analyzed for a bubble of radius $a = 100$ μm. In Supplementary Figure 3B we show that by increasing the driving signal by increments

of 10 mVpp (1 mVpp), the bubble displacement of the bubble is 200 μm (50 μm) with typical oscillations of 10 μm around the equilibrium position.

Acoustic streaming The acoustic streaming reaches a steady state after tens of milliseconds and is readily observable by tracking the motion of any small object floating in the vicinity of the flow. The trajectories of a few natural impurities, present in the propagation medium, and of size smaller than 2 μm are shown in supplementary Figure 4 A, for $p_0 = 0.7$ MPa and a duty cycle equal to 44%. In the absence of ultrasound, these particles have a low density contrast with the fluid bulk and can therefore serve to assess an approximate value of the streaming flow velocity, u_s. In Figure 4B we plot the vertical particle velocity, v_z, of 5 selected tracers circulating near the beam focus, $(x, z) = (0, 0)$. We estimate the streaming flow velocity to be near $u_s = v_z \sim 4.5$ mm/s which is lower than the value $v_z = 8$ mm/s necessary to retrieve the total pushing force measured for small bubbles including a Stokes' drag (Fig.2 B, dashed-curve). The remaining discrepancy may arise from the non-ideal geometry of the tracers used in the evaluation of u_s, but also from an additional dissipation of the acoustic momentum flux in the boundary layer surrounding the bubble surface (6).

Experimental measurement of the far-field scattering diagram The far-field scattering function, $f_s(\theta, \varphi)$ (Equation [3]), is axisymmetric for bubbles located on the propagation axis ($f_s(\theta, \varphi) = f_s(\theta)$). The magnitude of the scattering coefficients R_n and polar plots of the scattering form function are shown in Supplementary Figure 5 (B-C). For small bubbles, the dominant scattering modes are monopolar and dipolar. However, the broken symmetry induced by the helicoidal nature of the wavefronts excludes monopolar oscillations for on-axis bubbles (See discussion above). This explains the dominant dipolar oscillation for a bubble of radius $a = 50$ μm, Supplementary Fig. 5B). For a bubble of radius $a = 270$ μm, the perimeter becomes comparable to the acoustic wavelength and higher order multipoles can be excited in the so-called Mie scattering regime. The bubble tends to scatter predominantly in the bubble rear ($\theta < 90°$). To measure this enhanced scattering we exploited the good positional stability of the trap and the slow dissolution rate of large bubbles ($a > 200$) that would not significantly shrink over a course of a few minutes. It was thus possible to scan the scattered pressure field using a hydrophone (Supplementary Fig.5 D). Nevertheless, in order to isolate the very weak echo emitted by a single microbubble, predicted to be more than 2 orders of magnitude lower than the incident pressure field, several precautions were taken. First, the hydrophone was positioned in the far field, approximately 25 mm away from the bubble centre. The tone burst was reduced to 5 acoustic cycles in order to distinguish the bubble echo from other direct arrivals from the acoustic transducer or reflections in the water tank. The total propagation time of the wave scattered by the bubble towards the hydrophone was estimated to be near 50 μs. It was hence necessary to retain a long 100 μs pulse repetition period. Despite the resulting 2% duty cycle for the excitation pattern, we secured conditions for which a sufficient trapping force was obtained by increasing the incident trap pressure to $p_0 = 2.25$ MPa. The echos detected from a single bubble of radius $a = 270$ μm are shown in Supplementary Figure 5 E, for several positions of the hydrophone, either on the bubble front or rare. From the amplitude of the echos we extracted a measure of the scattered field ($p_s \sim 5$ kPa). The distance R to the bubble centre was calculated from the time of arrival of each echo. Using Eq.[3], we computed the experimental far-field scattering functions shown in Fig.2 C.

3. Position-controlled bubble dynamics in the presence of a wall

To analyze the bubble dynamics of an isolated bubble, we combined the acoustical trap setup with a secondary source of ultrasound (Hesentec) driving the oxygen bubbles with the pressure change $p' = p'_0 \sin(2\pi f' t)$ at a lower frequency $f' = 28.2$ kHz. If the pressure field associated with the low-frequency wave is strong, or the bubble close to its resonant size ($a_M = 115$ μm at f'), the vertical trapping position z is affected by an additional acoustic force arising from the bubble volume oscillations forced by the low-frequency field (Eq.[1] in the main text). We observed that the bubble was attracted towards the boundary when it was larger than its resonant size. However, in most situations the primary trap remained stronger and a

readjustment of the trapping power allows to move the bubble back to its initial position. Conversely, if the bubble is smaller than resonance, it is pushed away from the wall by the combined effect of the pushing beam and the secondary force. This could either result in the establishment of a new equilibrium position further down the trap, but also to the loss of the trapped bubble.

The high speed camera was set to acquire 300,000 frames per second. The induced radius oscillations, $R(t) = a(1 + \frac{\Delta a}{a}\sin(2\pi f' \mathrm{t} + \Phi)$ are extracted from the raw videos by subtracting a background image to each frame, applying a binary threshold and computing the bubble area. The acquisition of an initial background image with the bubble removed is particularly important in the presence of a wall that projects a shadow on the side-view images. An example of an oscillating bubble radius with time is shown in Supplementary Figure 6 A. From the Fourier transform of $R(t)$ we can extract the oscillation magnitude Δa at the driving frequency f' (Supplementary Fig.6 B). It is also seen that the oscillations are linear under the sufficiently low driving pressure amplitude. The average radius a decreases overtime driven by the excess Laplace pressure inside the bubble. It is hence possible to perform successive measurements of the bubble dynamics with a constant forcing and frequency, but for a bubble of changing size (Supplementary Fig.6 B). Examples of reconstructed resonance curves are shown in Supplementary Fig.6 D and Figure 3 B.

Free bubbles For small amplitude radial oscillations $x = \Delta a/a << 1$ of a freely suspended bubble, the dynamics can be compared with that of a one-dimensional damped harmonic oscillator (13):

$$\ddot{x} + 2\beta\dot{x} + \omega_0^2 x = -p_0' \sin(2\pi f' t)/\rho a^2. \quad [14]$$

The resonant frequency is given by $\omega_R^2 = \omega_0^2(1 - \frac{2\beta^2}{\omega_0^2})$ where $\omega_0 = (\alpha p_A/\rho a^2)^{1/2}$ is the natural undamped oscillation frequency, α is the polytropic coefficient and $p_A = 10^5$ Pa the ambient pressure. The expression for the natural oscillation frequency was originally obtained by Minnaert, valid for sufficiently large bubbles for which surface tension effects are negligible. Equivalently, we can define the Minnaert resonance radius, $a_M = \frac{1}{2\pi f'}(\alpha p_A/\rho)^{1/2}$ related to our secondary sound wave of frequency, f', that supposes an adiabatic gas expansion process ($\alpha = \gamma$) where γ is the specif heat ratio. a_M was used to normalize the experimental curves and fits in Fig.4 B and Supplementary Fig.6 D. The total damping coefficient β incorporates the dissipation occurring in the process of thermal attenuation in the bubble core, acoustic wave radiation and viscous dissipation in the fluid bulk (13). The thermal losses are mainly responsible for the damping in the range of bubble radii investigated here (see Supplementary Fig.6 E.). Therefore, in fitting the measured data, we hence assume three independent fitting parameters: the "thermal viscosity" introduced by Prosperetti, μ_{th} related to the oscillator damping as $\beta = \frac{2\mu_{th}}{\rho a^2}$, the natural frequency ω_0 and the driving acoustic pressure, p_0' of the secondary source of ultrasound. We make a step further in simplifying the problem by assuming that μ_{th} is radius independent and takes as average value $\mu_{th} = 0.038$ Pa·s (Supplementary Fig.6 F). In doing so, we focus on determining an average damping coefficient without identifying the exact nature of the thermal mechanisms at play, which is sufficient for the main scope of detecting the influence of an adjacent wall. From the fit we obtain respectively $\omega_0/\omega_0^{exp} = 99$ %, $\mu_{th}/\mu_{th}^{exp} = 73$ % and $p_0'^{exp} = 760$ Pa for a bubble isolated in the bulk using the trapping setup (we had no initial pressure calibration for the secondary acoustic source). Equivalently, the fitted resonance radius is very close to the theoretical value obtained by Minnaert. However, according to Prosperetti's model, it was expected to observe a 4% shift towards a lower resonant radius with the correct calculation of the polytropic coefficient and including the thermal damping process. The damping also reads a higher value β^{exp} relative to the model. Both observations possibly find an explanation in the rapid contamination of the bubble interface that can support additional elasticity and higher dissipation.

Presence of a wall The effect of an adjacent wall on bubble dynamics is still matter of debate, primarily because of the scarce experimental data available, complications owed to bubble encapsulations, and limited efforts to model the behavior of the bubble near a wall. In particular, to our knowledge, the work of Doinikov *et al* is the only one to consider a compliant boundary (14). In the linear regime, the resonance frequency for a bubble near a wall, ω_w, is found to be affected as $\omega_w^2 = \omega_0^2/\epsilon$, where for soft boundaries ϵ is

a function of the separation distance between the bubble and the wall, the density and the thickness of the elastic layer. Computing the resonance radius following Doinikov *et al* with our experimental settings, we do find an approximate 1% shift of the resonant radius relative to the unbounded free bubble case, but towards the smaller radii which is in contradiction with the shifts shown in Fig.4 B, where we find $a/a_M = 1.007$ and 1.015% for the 3 and 4% agarose gels respectively. A limitation in the theoretical work we referred to is to consider small bubbles compared to the separation distance, $a/d << 1$. Here, the resonance radius is such that $a \sim d$ which seems to have additional implications on the bubble dynamics.

Supplementary Information

Supplementary Video 1: Microbubble single-beam trap

Supplementary Video 2: 3D manipulation of a single microbubble

Supplementary Video 3: Microbubble manipulation in crowded environments

Supplementary Video 4: Position-controlled microbubble dynamics with an acoustic trap

Supplementary Video 5: Payload release from nanoparticle-coated microbubbles

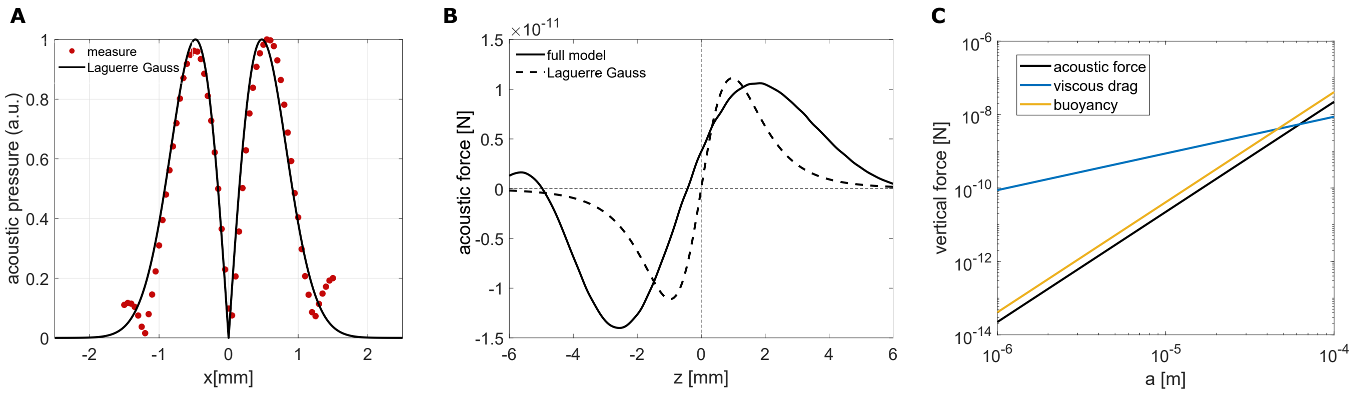

Fig.S 1. Force model for small bubbles. (**A**) Pressure field approximation with a Laguerre-Gauss (LG) vortex mode. (**B**) Comparison of the acoustic force with the full model and the LG model for a bubble with radius $a = 5$ μm. (**B**) Change of the acoustic force, viscous drag an buoyancy with the bubble radius.

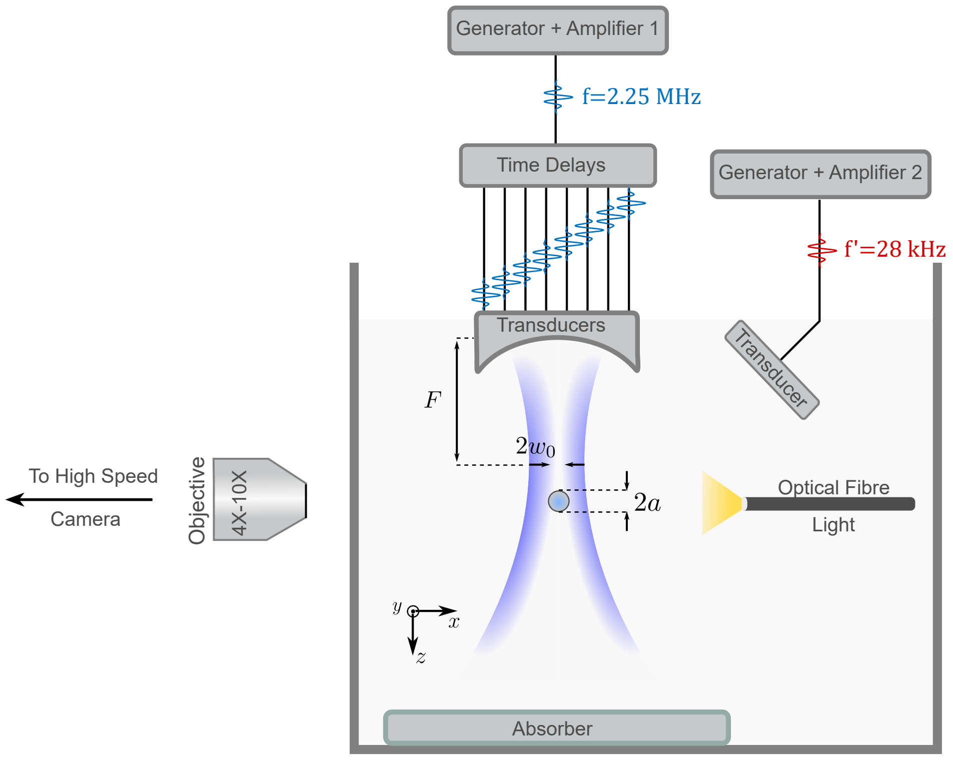

Fig.S 2. Experimental setup. The experimental setup consists of an 8-element focused (F/D=1) ultrasonic source emitting a vortex beam in a tank filled with water. The source is driven by an electronic delay module introducing the desired phase shifts to a monochromatic burst ($f = 2.25$ MHz) generated by a signal generator and a first amplifier. $F = 38$ mm is the focal length and trap working distance, $2w_0 = 1.2$ mm is the vortex ring diameter at the focus. A second low-frequency transducer ($f' = 28$ kHz) can be activated with an independent signal generator and amplifier to force oscillations of the trapped bubble. All the observations are made from a side view through a variable magnification long working distance objective and recorded with a high-speed camera. A fibre optic light is immersed in the water tank.

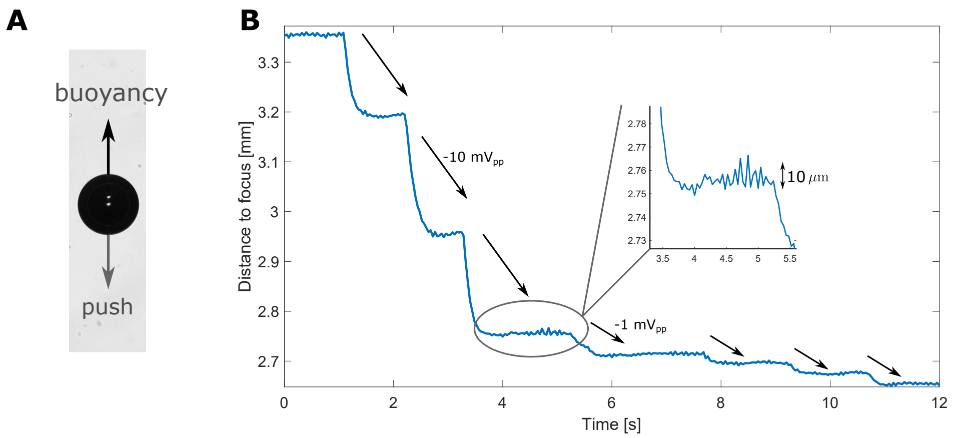

Fig.S 3. Axial manipulation precision. (**A**) The bubble's relative position to the acoustic focus can be adjusted by changing the driving amplitude of the beam's pushing force. The bubble radius is $a = 100$ μm. (**B**) An increment of 10 mVpp (1 mVpp) in the driving voltage leads to a 200 μm (50 μm) vertical shift. The typical center of mass fluctuations are 10 μm.

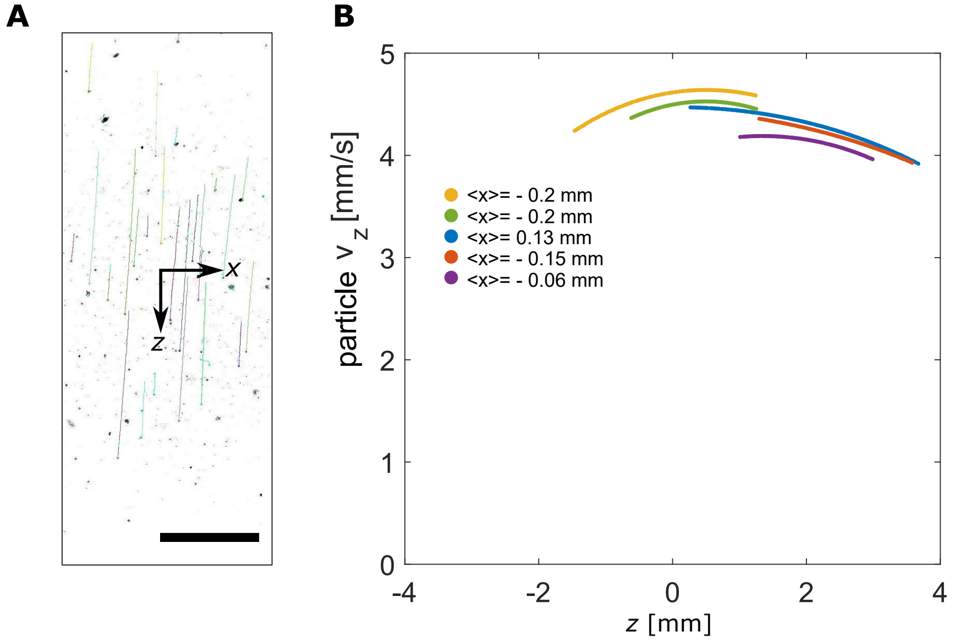

Fig.S 4. Acoustic streaming. (**A**) Selected trajectories of unbouyant particles of size $a < 2$ μm near the acoustic focus $(x, z) = (0, 0)$. (**B**) Vertical particle velocity, v_z of 5 selected trajectories circulating at an average lateral distance $\langle x \rangle < 200$ μm from the propagation axis. Scale bar length: 500 μm.

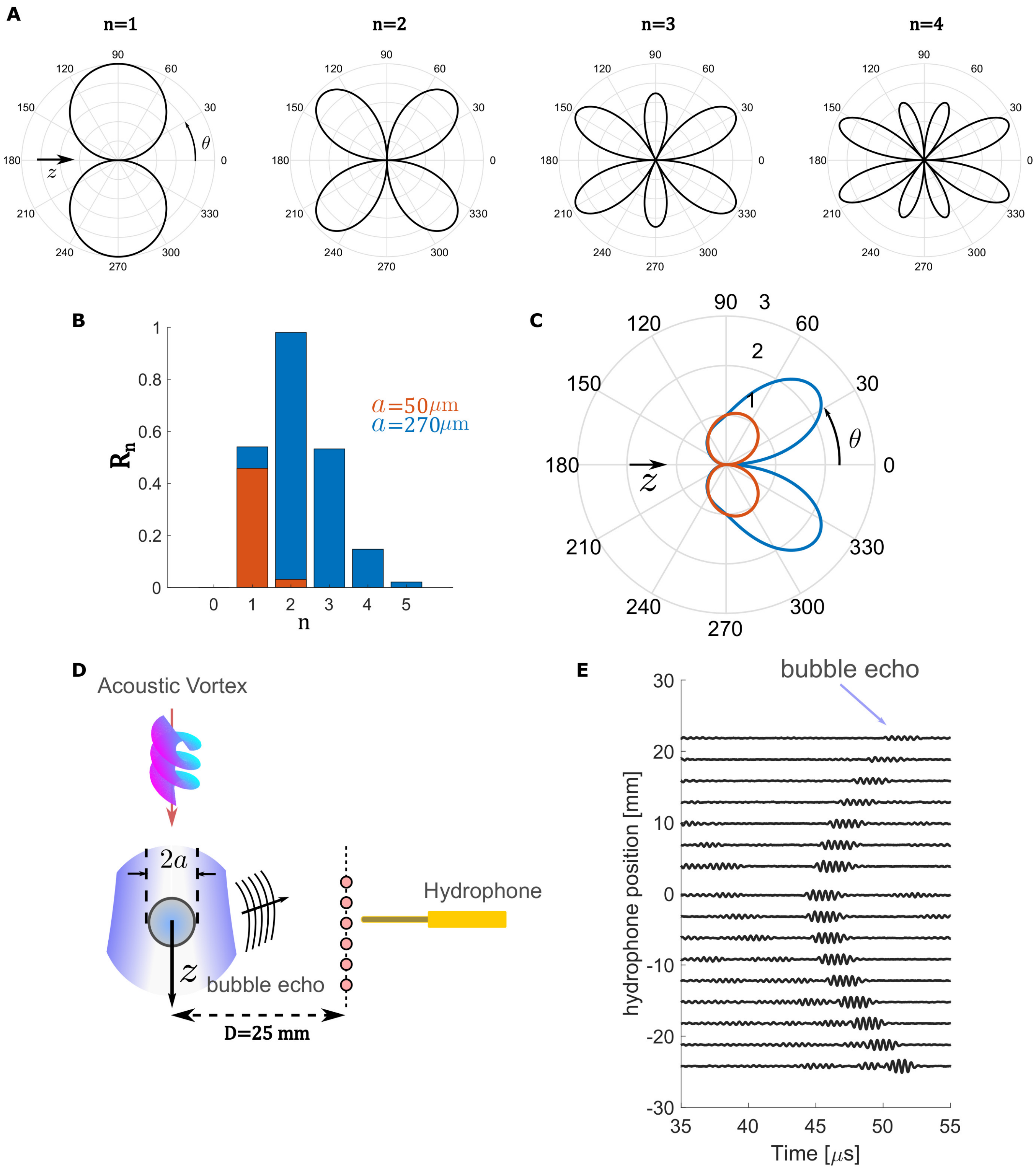

Fig.S 5. Acoustic field scattered by an isolated bubble. (**A**) Mutipolar modes decomposition of the scattered field: dipolar ($n = 1$), quadrupolar ($n = 2$), etc. (**B**) Amplitude of the complex scattering coefficients R_n for two different bubbles. (**C**) Small bubbles behave as dipoles whereas larger bubbles involve higher order modes and scatter predominantly in the bubble rare. (**D**) Sketch of the scattered field measurement protocol. 5 cycles of a helicoidal tone burst are scattered by the bubble and measured in the far-field by the hydrophone positioned more than 25 mm from the bubble centre. (**E**) Time traces of the isolated single-bubble scattering events.

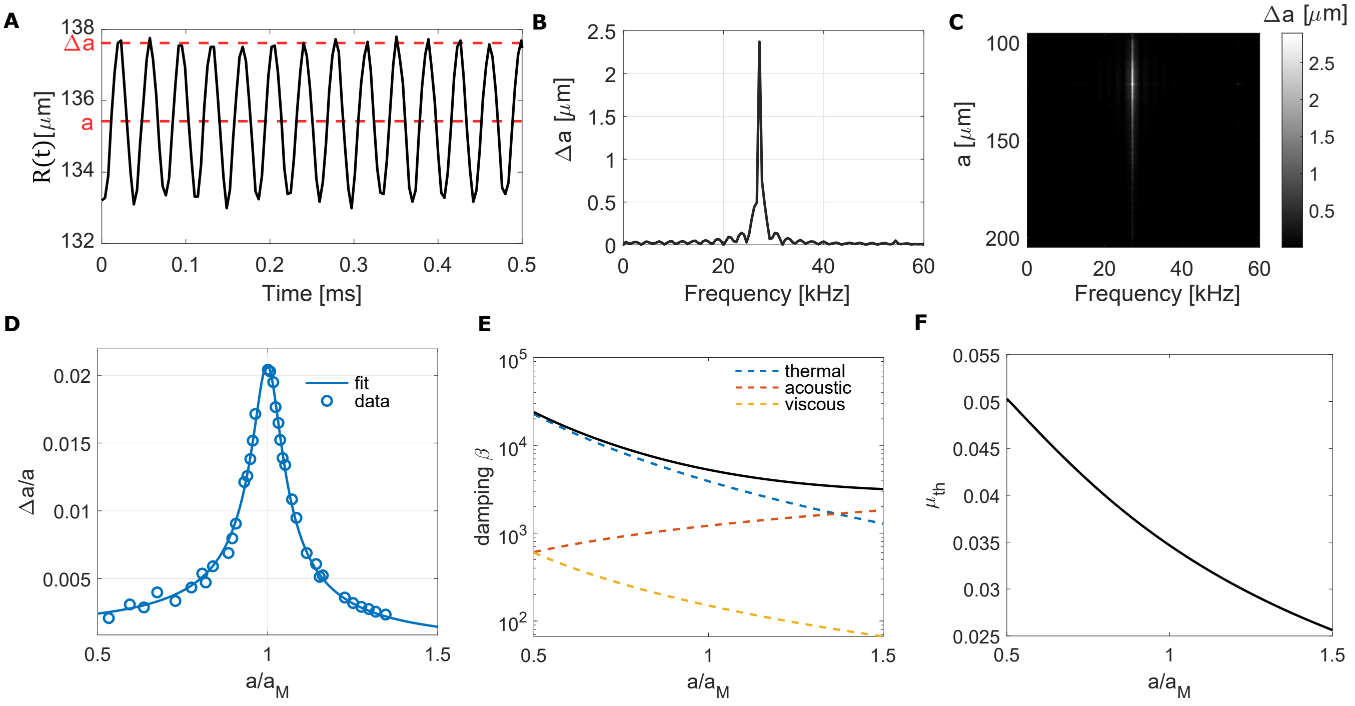

Fig.S 6. Isolated bubble resonance curve excited by a secondary source of ultrasound. (**A**) Time evolution of the bubble radius trapped by the tweezers setup and excited by a secondary source of ultrasound. (**B**) Fourier transform of the curve in **A**. (**C**) Same as (**B**) for all the subsequent bubble sizes at rest a that fall in between 50 and 150 μm for a bubble shrinking by dissolution. (**D**) Free bubble's resonance curve constructed from the bubble dynamics measured in the frequency spectrum in (**D**) at the driving frequency $f' = 28.2$ kHz. The black curve is obtained with a linear oscillator model for the dynamics (Methods). The blue curve is the best-fit to the data points. The curves are normalized by the theoretical Minnaert resonance frequency, $a_M = 115$ μm. (**E**) Damping coefficient β of the oscillator and its three main contributions. (**E**) Computed "thermal viscosity" in the investigated bubble size range.

1. D Baresch, JL Thomas, R Marchiano, Three-dimensional acoustic radiation force on an arbitrarily located elastic sphere. *The Journal Acoustical Society America* **133**, 25–36 (2013).
2. D Baresch, JL Thomas, R Marchiano, Spherical vortex beams of high radial degree for enhanced single-beam tweezers. *Journal Applied Physics* **113**, 184901 (2013).
3. C Feuillade, C Clay, Anderson (1950) revisited. *The Journal Acoustical Society America* **106**, 553–564 (1999).
4. D Baresch, JL Thomas, R Marchiano, Observation of a single-beam gradient force acoustical trap for elastic particles: acoustical tweezers. *Physical review letters* **116**, 024301 (2016).
5. D Baresch, Ph.D. thesis (Pierre et Marie Curie University) (2014).
6. JT Karlsen, H Bruus, Forces acting on a small particle in an acoustical field in a thermoviscous fluid. *Physical Review E* **92**, 043010 (2015).
7. LA Crum, AI Eller, Motion of bubbles in a stationary sound field. *The Journal Acoustical Society America* **48**, 181–189 (1970).
8. JF Nye, MV Berry, Dislocations in wave trains. *Proceedings Royal Society London. A. Mathematical Physical Sciences* **336**, 165–190 (1974).
9. L Allen, MW Beijersbergen, R Spreeuw, J Woerdman, Orbital angular momentum of light and the transformation of laguerre-gaussian laser modes. *Physical review A* **45**, 8185 (1992).
10. R Marchiano, JL Thomas, Synthesis and analysis of linear and nonlinear acoustical vortices. *Physical Review E* **71**, 066616 (2005).
11. A Anhäuser, R Wunenburger, E Brasselet, Acoustic rotational manipulation using orbital angular momentum transfer. *Physical review letters* **109**, 034301 (2012).
12. C Eckart, Vortices and streams caused by sound waves. *Physical review* **73**, 68 (1948).
13. A Prosperetti, Thermal effects and damping mechanisms in the forced radial oscillations of gas bubbles in liquids. *The Journal Acoustical Society America* **61**, 17–27 (1977).
14. AA Doinikov, L Aired, A Bouakaz, Acoustic scattering from a contrast agent microbubble near an elastic wall of finite thickness. *Physics Medicine & Biology* **56**, 6951 (2011).
